# Supplementary material for: Evolutionarily informed gene sets reveal conserved and lineage-modified transcriptional programs during vertebrate forebrain evolution
Source: bioRxiv. 2026 Mar 25:2026.03.24.713832. Preprint. [Version 1] doi: 10.64898/2026.03.24.713832 (PMC13041851; doi:10.64898/2026.03.24.713832)
Supplement: Supplement 1 [file media-1.zip › Supplementary_Data_Files/Description_of_Additional_Supplementary_Data_Files.docx]

**Description of Additional Supplementary Data Files**

File Name: Supplementary Data File 1

Description: Annotation of scGENUS-derived gene sets. Each gene set is assigned a unique identifier (gene-set ID) and associated with graph partitioning resolution(s) used for its identification (resolutions), the number of genes within the set (gene count), and corresponding gene symbols (gene). Gene symbols are provided separately for each species, with each sheet representing one species.

File Name: Supplementary Data File 2

Description: Results of differential gene-set expression testing used to identify cell-type-specific gene sets. Each sheet corresponds to one cell type and reports Wilcoxon rank-sum test statistics for each gene set, including p-value, average log2 fold change, percentage of cells expressing the gene set in the given cell type and in all other cell types, adjusted p-value, and the difference in these percentages.

File Name: Supplementary Data File 3

Description: Cell-type metadata for each species included in this study. Each sheet corresponds to one species and reports the original cell cluster annotation (Original_annotation), primary cell class (Class), secondary cell classification (Subclass), harmonized cell-type annotation (Celltype), anatomical region annotation (Anatomical_region), and developmental origin classification of GABAergic interneuron subtypes (Interneuron_subtype). Harmonized cell-type annotations were used exclusively for reconstruction of cell-type phylogenies.

File Name: Supplementary Data File 4

Description: Metadata for Milo-derived KNN graph neighborhoods used in cross-species compositional analysis. Each row corresponds to a neighborhood. The columns x and y represent UMAP coordinates for visualization of the neighborhood graph. Columns corresponding to species (Amex, *Ambystoma mexicanum*; Ca, *Carassius auratus*; Cpbellii, *Chrysemys picta bellii*; Dr, *Danio rerio*; Gg, *Gallus gallus*; Hs, *Homo sapiens*; Mm, *Mus musculus*; Mz, *Maylandia zebra*; PetMar, *Petromyzon marinus*; Pv, *Pogona vitticeps*; Tgu, *Taeniopygia guttata*) report normalized species enrichment within each neighborhood, weighted by global species frequencies. The column entropy_scaled represents the Shannon entropy of species composition within each neighborhood, quantifying the degree of cross-species mixing. The column radius is proportional to the number of cells contained in each neighborhood. The column km_cluster indicates k-means cluster assignments of neighborhoods based on their graph representation.

File Name: Supplementary Data File 5

Description: Gene sets exhibiting state- and species-dependent expression dynamics along radial glial developmental trajectories. Sheet 1 reports results of state-dependent analysis testing for differences in gene-set expression dynamics between neurogenic and gliogenic branches using natural spline regression models. Sheets 2 and 3 report results of species-specific analyses of gene-set expression dynamics along pseudotime within the neurogenic and gliogenic branches, respectively, based on generalized linear models implemented in Monocle3, where species-pseudotime interaction terms capture species-dependent differences in expression dynamics.

File Name: Supplementary Data File 6

Description: File Name: Supplementary Data File 5

Description: Results of MAGMA gene-set enrichment analysis testing associations between scGENUS-derived gene sets and human cognitive traits and neuropsychiatric disorders. Each row corresponds to one gene set–trait pair and reports the trait, enrichment statistics, and standardized effect size.
