# Supplementary material for: Evolutionarily informed gene sets reveal conserved and lineage-modified transcriptional programs during vertebrate forebrain evolution
Source: bioRxiv. 2026 Mar 25:2026.03.24.713832. Preprint. [Version 1] doi: 10.64898/2026.03.24.713832 (PMC13041851; doi:10.64898/2026.03.24.713832)
Supplement: Supplement 1 [file media-1.zip › Supplementary_Data_Files/Manuscript_HHE_supp_03232026.docx]

**SUPPLEMENT**

**FIGURES**

**Figure S1. Resolution-dependent filtering of evolutionarily informed gene sets.** Proportion of gene sets containing genes from all eleven vertebrate species as a function of Leiden clustering resolution applied to the global gene homology graph. At low to intermediate resolutions (0.01-10), a substantial fraction of gene sets (greater than 50%) span all species, indicating the presence of broadly conserved gene families coexisting with lineage-restricted sets. At higher resolutions (greater than 10), the gene-set composition becomes increasingly dominated by lineage-restricted gene sets, and the proportion of gene sets spanning all species correspondingly decreases. This analysis guided the selection of clustering resolutions and downstream filtering to balance cross-species representation with gene-set specificity and interpretability.

**Figure S2. Gene-set-based low-dimensional embeddings of single-cell transcriptomes across vertebrate species.** (A) UMAP embeddings of gene-set-level single-cell profiles for each vertebrate species, colored by primary cell class (neuronal, glial, and non-neural). Major cell classes form coherent and separable clusters within and across species, indicating that gene-set-based features preserve broad cellular organization. (B) UMAP embeddings of the same gene-set-level single-cell profiles colored by secondary cell class, including major neuronal and glial subtypes. Finer-grained cell-type structure is retained within each species, indicating that gene-set-based representations capture biologically meaningful cellular heterogeneity while enabling cross-species comparison.

**Figure S3. Gene-set-level differential expression identifies species-specific gene sets within vertebrate forebrain cell types.** Dot plots show species-specific scGENUS gene sets identified in (A) GABAergic neurons; (B) glutamatergic neurons; (C) vascular cells; (D) microglia; (E) radial glia; (F) astrocytes; (G) ependymal cells; (H) oligodendrocytes; (I) oligodendrocyte precursor cells (OPCs); (J) immune cells. Gene-set identifiers are listed on the left; representative human gene symbols within each gene set are shown on the right to facilitate annotation. Asterisks (*) denote gene sets lacking a human ortholog, in which case representative locus identifiers or species-specific gene names are displayed. Dot size indicates the fraction of cells expressing each gene set, and color represents scaled mean expression.

**Figure S4. Distribution of local species mixing indices across Milo-defined neighborhoods.** (A) k-means clustering of Milo-defined transcriptional neighborhoods. Each node represents a local neighborhood in the scGENUS gene-set space; and clusters group neighborhoods with similar species composition profiles. (B) Distribution of the local species mixing index across neighborhoods. Clusters with lower median local species mixing index correspond to lineage-restricted or species-biased neighborhoods highlighted in Fig. 2E.

**Figure S5. Sublineage-restricted analysis of evolutionary cell state divergence.** (A) Histogram of log10-transformed L1 distances between centroids of human and mouse within each cell type (evolution-induced cell state shifts) versus pairwise L1 distances between centroids of distinct cell types (cell type shifts) within mammalian species. (B) Same as in (A), restricted to reptilian species (lizard and turtle). (C) Same as in (A), restricted to avian species (songbird and chicken).

**Figure S6. Phylogenetic organization of glutamatergic, glial, and non-neural forebrain cell types.** (A) Unrooted, distance-based phylogeny of glutamatergic neuronal subtypes inferred from transcriptomic similarity in the shared PCA space derived from scGENUS gene-set expression (first 50 principal components). Subtypes from eleven vertebrate species are shown at the tips. Branch lengths reflect transcriptomic divergence. Major anatomical subdivisions are indicated. Abbreviations: D, area dorsalis; V, area ventralis; Dc, large-celled subdivision of Dm; Vsst, ventral Sst; Ppa, nucleus preopticus parvocellularis anterioris; a, anterior; p, posterior; d, dorsal; v, ventral; m, medial; l, lateral; MC, medial cortex; DMC, dorsomedial cortex; pDC and aDC, posterior and anterior dorsal cortex; PT, pallial thickening; aLC and pLC, anterior and posterior lateral cortex; aDVR and pDVR, anterior and posterior dorsal ventricular ridge; DG, dentate gyrus; CA, cornu ammonis; OB, olfactory bulb; NAC, nucleus accumbens neurons. Tip color represents the leaf stability index (LSI) calculated from 1,000 bootstrap resampling iterations, with values ranging from 0 (low stability) to 1 (high stability). (B) Same as in (A), for glial populations (radial glia, astrocytes, ependymal cells, OPCs, oligodendrocytes, and microglia). (C) Same as in (A), for non-neural populations (vascular and peripheral immune cell types).

**Figure S7. Gene-set expression conservation is independent of gene-set size and functional enrichment of highly conserved gene sets.** (A) Distribution of gene-set expression conservation scores grouped by gene-set size. (B) Biological pathway enrichment results of the 1,086 highly conserved gene sets (based on human orthologs). Circle size indicates the number of genes associated with each GO term, and color represents FDR-adjusted *p*-values.

**Figure S8. TGFB-enriched gene-set density and GO enrichment of dynamic modules along neurogenic and gliogenic branches.** (A) Gene-set-weighted density estimates for TGFB1-3 ligand-associated signaling projected onto the UMAP embedding. (B, C) Gene Ontology (GO) enrichment analysis of gene-set modules dynamically expressed along the neurogenic (B) and gliogenic (C) branches. Bars represent -log_10_(adjusted *p*-values).

**Figure S9. Functional enrichment and human cell-type localization of neuropsychiatric-associated gene sets.** (A) Functional enrichment analysis of gene sets showing significant enrichment in MAGMA analysis. Circle size indicates the number of genes associated with each GO term, and color represents -log_10_(adjusted *p*-values). (B) scGENUS-derived UMAP embedding of human prefrontal cortex, colored by cell type annotations. (C, D) Projection of SCZ- and BPD-associated gene-set expression onto the same scGENUS-derived UMAP embedding of human prefrontal cortex. Module scores are shown for SCZ-associated gene sets (C) and BPD-associated gene sets (D).
